# Targeting MET in 2025: From Exon 14 Skipping to MET-Amplified Acquired Resistance in Non-Small Cell Lung Cancer

**DOI:** 10.3390/ijms27135883

**Published:** 2026-06-30

**Authors:** Aliya Khan, Michael Imeh, Priyanka Barad, Daniel Rosas

**Affiliations:** 1 Hematology-Oncology Fellowship Program, Memorial Cancer Institute, Memorial Healthcare System, Hollywood, FL 33021, USA; akhan@mhs.net (A.K.); mimeh@mhs.net (M.I.); 2 Harnett Health Internal Medicine Residency, Dunn, NC 28334, USA; pbarad23@gmail.com

**Keywords:** MET, non-small cell lung cancer, MET exon 14 skipping, MET amplification, capmatinib, tepotinib, telisotuzumab vedotin, osimertinib resistance, targeted therapy, precision oncology

## Abstract

MET pathway alterations have evolved from a niche translational interest into one of the most clinically actionable axes in non-small cell lung cancer (NSCLC). Three biologically distinct lesions—MET exon 14 (METex14) skipping mutations, focal high-level MET amplification, and c-Met protein overexpression—are now individually targetable, each with its own diagnostic prerequisites and therapeutic class. Selective type Ib MET tyrosine kinase inhibitors (capmatinib, tepotinib) anchor first-line therapy for METex14, while next-generation agents and type II inhibitors are being developed to address on-target D1228 and Y1230 resistance mutations. In parallel, MET amplification has emerged as a leading mechanism of acquired resistance to osimertinib in EGFR-mutated NSCLC, with the SAVANNAH, SACHI, and INSIGHT 2 trials providing biomarker-guided combination strategies. The 2025 accelerated approval of telisotuzumab vedotin for c-Met-overexpressing tumors expanded the therapeutic armamentarium beyond kinase inhibition. Despite these advances, lineage plasticity, polyclonal bypass signaling, and inconsistent diagnostic thresholds for MET amplification continue to limit durable benefit. This review integrates the molecular biology, current clinical evidence, resistance mechanisms, and a proposed 2025 treatment algorithm for MET-altered NSCLC, with emphasis on the translational interface between mutation class, drug class, and emerging combinatorial approaches. As a narrative review, it synthesizes peer-reviewed literature and pivotal trial and regulatory data through early 2026, identified by structured searches of PubMed and major oncology congress proceedings, and prioritizes sources that link mutation class to drug class and resistance mechanism.

## 1. Introduction

Lung cancer remains the foremost cause of cancer-related death worldwide. In 2022, approximately 2.5 million individuals were diagnosed with lung cancer globally, corresponding to an age-standardized rate of 23.6 cases per 100,000 persons [1,2]. With nearly 1.8 million deaths annually, lung cancer accounts for the largest proportion of cancer mortality worldwide, and the five-year survival rate in most countries remains below 20% [1,2]. Non-small cell lung cancer (NSCLC) constitutes the dominant histological category, comprising over 80% of all diagnosed lung cancers [3]. Notably, lung adenocarcinoma has emerged as the predominant subtype in recent years, with increasing incidence among younger generations, particularly females, in most countries assessed [3].

The past two decades have witnessed a paradigm shift in NSCLC management, driven by the identification and therapeutic exploitation of oncogenic driver alterations. Targetable drivers now include mutations in epidermal growth factor receptor (EGFR) and BRAF, as well as fusions in anaplastic lymphoma kinase (ALK), ROS1, neurotrophic receptor tyrosine kinase (NTRK), and rearranged during transfection (RET) genes, each amenable to selective tyrosine kinase inhibitors (TKIs) [4]. The increased adoption of comprehensive molecular profiling and actionable genomic alterations now informs treatment strategies across both early and advanced disease settings [4]. Within this genomically stratified landscape, MET (the official HGNC symbol; the gene encodes a receptor tyrosine kinase and the acronym derives from the MNNG-HOS transforming gene, the long-used expansion “mesenchymal–epithelial transition factor” being a recognized misnomer [5]) has emerged as a clinically significant and therapeutically actionable target [6,7,8].

MET encodes a receptor tyrosine kinase whose principal ligand is hepatocyte growth factor (HGF). Binding of HGF to MET initiates intracellular signaling cascades that regulate embryogenesis and wound healing in normal cells; however, in the oncogenic context, aberrant HGF/MET axis activation driven by gene mutations, overexpression, and amplification promotes tumor development and progression through the phosphoinositide 3-kinase/AKT (PI3K/AKT), Ras/mitogen-activated protein kinase (RAS/MAPK), JAK/signal transducer and activator of transcription (STAT), and Wnt/β-catenin pathways [7,9]. MET dysregulation in NSCLC assumes two principal forms: MET exon 14 (METex14) skipping mutations, which serve as an independent oncogenic driver, and MET amplification (METamp), which may occur as a primary alteration or as a mechanism of acquired resistance to other targeted therapies [6,10].

Median METex14 skipping frequency is approximately 2.0% in unselected NSCLC, with minimal geographic variation, rising to 12% in sarcomatoid histology [6,11]. MET amplification occurs in approximately 1–6% of patients with NSCLC as a primary event; however, it represents an important acquired resistance mechanism to first- or second-generation EGFR-TKIs, accounting for approximately 10–20% of resistance cases in EGFR-mutant NSCLC [10,12]. Secondary MET amplification occurs in approximately 15% of cases previously treated with an EGFR inhibitor [10].

The clinical development of MET-targeting strategies has evolved considerably. Early efforts employing the monovalent monoclonal antibody onartuzumab, which blocks HGF–MET interaction, failed to demonstrate meaningful efficacy in the phase III METLung trial, partly due to reliance on immunohistochemistry (IHC)-based patient selection without genomic stratification [13]. The METLung trial, which examined onartuzumab plus erlotinib in previously treated MET-positive NSCLC, did not meet its primary endpoint of improved overall survival [13]. The subsequent recognition that METex14 skipping confers oncogene addiction amenable to selective TKI inhibition represented a conceptual turning point [14,15]. Results from the GEOMETRY mono-1 and VISION phase II trials demonstrated significant clinical activity with capmatinib and tepotinib respectively [16,17], establishing METex14 skipping as a validated therapeutic target and supporting upfront genomic testing in all NSCLC patients.

The present review examines the molecular biology of MET alterations in NSCLC, with particular emphasis on METex14 skipping as a primary oncogenic driver and MET amplification both as a primary (de novo) oncogenic driver and as a mechanism of acquired resistance to EGFR-directed therapy. We discuss the diagnostic implications of each alteration, review the evidence base for approved MET inhibitors, and situate these agents within current 2025 treatment algorithms. Finally, we address emerging therapeutic strategies and the translational challenges that will define the next phase of MET-directed oncology. In contrast to prior MET reviews, our distinct contribution is to organize the field explicitly around a mutation-class → drug-class → resistance-mechanism framework and to integrate the most recent 2025 developments—the accelerated approval of telisotuzumab vedotin, the phase III SACHI readout and subsequent China approval of savolitinib plus osimertinib, and the maturation of type I/type II resistance biology—into a single, clinically oriented synthesis with an explicit treatment algorithm. This topic is particularly relevant for the readership of the International Journal of Molecular Sciences, as it represents an exemplar of how mechanistic understanding of receptor tyrosine kinase biology translates directly into precision therapeutic intervention.

## 2. MET Biology and Oncogenic Activation in NSCLC

### 2.1. Structure and Function of the MET Receptor Tyrosine Kinase

The MET proto-oncogene, located on chromosome 7q31, encodes a heterodimeric receptor tyrosine kinase expressed predominantly on epithelial cells of diverse organs including liver, pancreas, kidney, prostate, and bone marrow [7,9]. MET is a prototypal tyrosine kinase receptor that plays a key role in the interaction between mesenchyme and epithelia during embryogenesis and tissue homeostasis; its ligand HGF is produced by cells of mesenchymal origin and promotes migration and proliferation in epithelial target cells [9,18]. The mature MET protein consists of an extracellular α-chain harboring the ligand-binding domain and an intracellular β-chain containing the tyrosine kinase domain and a carboxy-terminal multifunctional docking site. The extracellular α-chain contains the ligand-binding pocket, while the intracellular β-chain contains the tyrosine kinase domain and a conserved two-tyrosine multifunctional docking site that interacts with multiple SRC homology 2 (SH2)-containing signal transducers [7,18].

**Figure 1:**
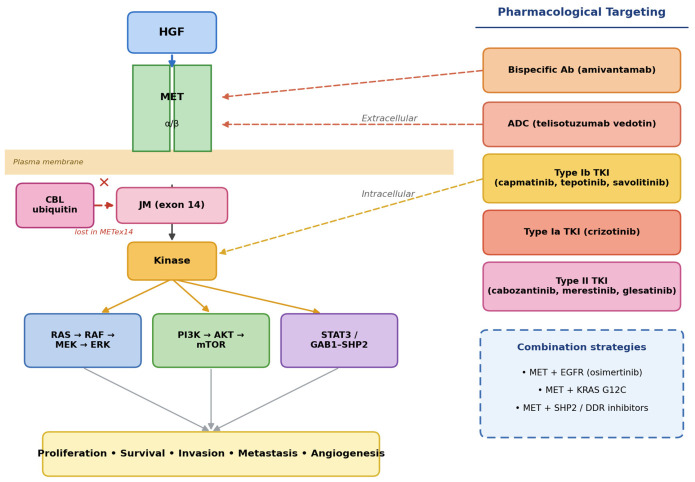
Schematic representation of MET receptor signaling and pharmacological points of intervention in non-small cell lung cancer. The figure depicts HGF-induced MET dimerization, the juxtamembrane region encoded by exon 14, and downstream RAS–MAPK, PI3K–AKT, and STAT3 signaling outputs. METex14 alterations disrupt CBL-mediated ubiquitination via loss of the Y1003 binding site, leading to receptor accumulation. Therapeutic agents are mapped to their mechanistic targets: bispecific antibodies (e.g., amivantamab) and antibody-drug conjugates (e.g., telisotuzumab vedotin) act at the extracellular domain; type Ia (crizotinib), type Ib (capmatinib, tepotinib, savolitinib), and type II (cabozantinib, merestinib) tyrosine kinase inhibitors act at the kinase domain. As drawn, the schematic emphasizes METex14 and HGF-dependent activation; MET amplification-driven signaling and EGFR–MET bypass interactions, although major themes of this review, are represented more schematically. STAT3 is shown as a transcriptional signaling output and is mechanistically distinct from the GAB1/SHP2 adaptor and scaffold proteins, which act as upstream signal mediators rather than equivalent downstream outputs. Of the combination strategies depicted, MET + EGFR inhibition is clinically validated in the post-osimertinib MET-amplified setting, whereas MET + KRAS G12C and MET + SHP2/DNA-damage-response-inhibitor combinations remain investigational. ADC, antibody-drug conjugate; HGF, hepatocyte growth factor; JM, juxtamembrane domain.

Upon HGF binding, MET undergoes autophosphorylation at tyrosine residues Y1234 and Y1235 within the kinase domain, followed by phosphorylation of docking site residues Y1349 and Y1356 in the carboxy-terminal tail, enabling downstream signal transduction through pathways governing cellular proliferation, survival, migration, invasion, angiogenesis, and epithelial-to-mesenchymal transition (EMT) [9,18]. The principal downstream effectors activated by the HGF/MET axis include PI3K/AKT, RAS/MAPK, and STAT3, whose combined activation stimulates cell growth and motility while inhibiting cell death, resulting in effective cell–cell dissociation, invasion into the extracellular matrix, and metastasis [9] (Figure 1). MET also engages cross-talk with other receptor tyrosine kinases, including EGFR, creating convergent signaling networks relevant to both primary oncogenesis and therapeutic resistance [9,19].

### 2.2. METex14 Skipping: Molecular Mechanism and Oncogenic Consequences

METex14 skipping represents the most prevalent oncogenic MET mutation class in NSCLC. Mutations leading to exon 14 skipping were first reported in 2003 in small-cell lung cancer and subsequently in NSCLC in 2005; more than 200 distinct genomic alterations have since been identified through comprehensive cancer genome profiling [6,20]. Molecular abnormalities in and around exon 14—including point mutations, insertions, and deletions—can disrupt essential consensus sequences such as splice acceptor and donor sites, the polypyrimidine tract, and branch sites, giving rise to this splicing variant and resulting in the loss of 141 base pairs and a 47-amino acid in-frame deletion within the juxtamembrane domain (JXD) [20,21].

The central oncogenic consequence of exon 14 skipping is impaired receptor degradation. Exon 14 encodes the juxtamembrane domain containing the Y1003 residue, which serves as a binding site for the E3 ubiquitin ligase CBL; skipping of exon 14 leads to decreased MET ubiquitination and degradation, increased MET protein stability, and amplified ligand-dependent downstream signaling [21,22]. In cells stably expressing METΔex14, HGF stimulation results in delayed degradation and oncogenic activation, and loss of the CBL-binding site has been established as the key event rendering METΔex14 oncogenic [21,22]. Importantly, upon HGF-mediated MET activation, the PI3K/AKT, RAS/MAPK, and STAT3 pathways are all engaged, and METΔex14 mutations usually occur in the absence of other known driver mutations such as EGFR, KRAS, or ALK [6,11].

Exon 14 also harbors a caspase cleavage site (ESVD1002), and its loss in METΔex14 may further impair apoptotic regulation, contributing to tumor cell survival under conditions of stress [22]. The molecular diversity of METex14 alterations—spanning splice donor, splice acceptor, polypyrimidine tract, and branch point mutations—poses substantial challenges for diagnostic assay design [6,20]. RNA-based next-generation sequencing (NGS) is the most effective method for detecting METex14 skipping mutations, as DNA-based sequencing may miss alterations in deep intronic regions that affect splicing [6,23,24]. DNA- and RNA-based NGS are nonetheless best regarded as complementary rather than mutually exclusive: DNA panels capture many canonical splice-site alterations but differ in intronic coverage, whereas RNA confirmation captures the functional skipping event itself. Concordance between the two is high but imperfect, and individual panel design and coverage limitations should be considered, particularly when a clinically suspected case tests negative on a single platform. Consistent with this, complementary DNA/RNA-NGS testing has been shown to increase the detection rate of METex14 and to uncover previously uncharacterized exonic splicing variants, reinforcing the value of an integrated DNA- and RNA-based diagnostic strategy [25].

### 2.3. MET Amplification: Primary Versus Secondary

MET amplification constitutes a molecularly and biologically distinct category of MET dysregulation. Primary (de novo) high-level MET amplification in treatment-naïve NSCLC behaves as an independent oncogenic driver and is largely mutually exclusive of other primary drivers (except MET mutation itself), whereas low-level amplification can co-occur with EGFR or KRAS mutations. This mutual exclusivity applies specifically to the primary setting: acquired high-level amplification arising under EGFR-TKI selective pressure characteristically coexists with the persistent original EGFR driver and is a bona fide resistance mechanism rather than an independent driver [10,11,26]. MET amplification can likewise co-occur with METex14 skipping and c-Met overexpression—reported METex14–amplification co-occurrence ranges widely across series (from negligible to approximately 40%), and stage IV METex14 tumors are enriched for concurrent amplification and high c-Met expression [27]. Focal, high-level amplification drives oncogene addiction and is associated with sensitivity to selective MET TKIs; copy-number gains below the threshold of high-level amplification (commonly defined as gene copy number ≥ 10 or MET/CEP7 ratio ≥ 2 by fluorescence in situ hybridization [FISH]) are less reliably predictive of TKI benefit and may represent polysomy rather than true gene amplification [10,28]. It should be emphasized that these cutoffs are not universally standardized: definitions of high-level amplification differ across trials and platforms (FISH versus NGS-based copy number), no single threshold is accepted across guidelines, and the same numerical value can carry different predictive weight depending on the assay and the clinical context (Section 7). Biologically, focal high-level amplification with a high MET/CEP7 ratio is the state most likely to reflect true MET dependency; a proportional increase in MET and centromere 7 signals instead indicates chromosome 7 polysomy rather than focal amplification and is a weaker predictor of TKI benefit, and clonal (truncal) amplification is more likely to drive dependency than subclonal or spatially heterogeneous gains. Current FISH and NGS thresholds therefore identify truly MET-dependent tumors only imperfectly: in routine practice, borderline or intermediate copy-number gains should be interpreted cautiously, ideally reconciled between FISH (with the explicit MET/CEP7 ratio) and quantitative NGS and weighed against the clinical context, rather than treated as definitively actionable.

In the context of EGFR-mutant NSCLC, MET amplification has attracted particular attention as a mechanism of acquired resistance. MET amplification is an important resistance mechanism to first- and second-generation EGFR-TKIs, and emerging preclinical and clinical evidence suggests it is also a key mechanism of acquired resistance to third-generation agents such as osimertinib, particularly when used as first-line therapy [10,12,29,30]. Secondary MET amplification occurs in approximately 15% of cases following EGFR TKI exposure, and promising results have been observed with combinations of MET TKIs and EGFR TKIs in this setting [10,31,32]. This interplay underscores the need for re-biopsy and liquid biopsy strategies at disease progression to identify actionable co-alterations [33,34].

### 2.4. MET Alterations in Tumor Heterogeneity and Metastatic Patterns

Patients with METex14 skipping NSCLC are typically elderly, with a median age of approximately 73 years, and adenocarcinoma histology predominates; unlike patients with EGFR mutations or ALK rearrangements, METex14 skipping shows an even distribution by sex and smoking status [6,11,35]. METex14 incidence varies markedly by histology, occurring in approximately 2% of adenocarcinomas, 1% of squamous cell carcinomas, 6% of adenosquamous carcinomas, and 13% of pulmonary sarcomatoid carcinomas (PSC) [6,11]. The enrichment of METex14 in PSC—a rare and historically chemotherapy-refractory histology—has significant therapeutic implications, as MET TKIs appear particularly active in this population [36].

The metastatic pattern of METex14 NSCLC exhibits distinct features. In a multicenter retrospective analysis of 148 patients with METΔex14 NSCLC who developed metastases, the most common sites were lymph nodes (67%), lung (53%), pleural/pericardial metastases or malignant effusions (51%), bone (49%), and brain (37%) [35]. The frequency of central nervous system (CNS) involvement—including leptomeningeal disease—in over one-third of patients underscores the clinical importance of CNS penetration for MET TKIs. In real-world data from the Italian ATLAS database, 24% of METex14 NSCLC patients presented with brain metastases, and intracranial responses were observed in 41% of evaluable patients treated with capmatinib or tepotinib, with an intracranial disease control rate of 76% [37].

Tumor heterogeneity in METex14 NSCLC is an emerging area of biological relevance. Novel molecular subtypes defined by co-occurring alterations—including MET amplification, TP53 mutations, and CDKN2A/B deletions—may identify subpopulations with differential sensitivity to MET TKIs [38]. METex14 NSCLC responds to MET TKIs with reported objective response rates ranging from 32% to 67.9% and median progression-free survival from 5.4 to 9.7 months, suggesting inter-tumoral heterogeneity that warrants further molecular characterization to guide more precise therapeutic strategies [6,39,40].

## 3. Therapeutic Landscape of MET Targeting in 2025

### 3.1. MET Tyrosine Kinase Inhibitors

#### 3.1.1. Type Ia and Type II—Multi-Kinase Inhibitors

Crizotinib, a first-generation type Ia ATP-competitive inhibitor with activity against ALK, ROS1, and MET, was historically the first agent demonstrated to be active in METex14-altered NSCLC. In the AcSé and PROFILE 1001 cohorts, ORRs of approximately 32% with median PFS of 7.3 months were observed [41]. Its limitations—visual disturbances, edema, hepatotoxicity, QTc prolongation, and inadequate CNS penetration—made it suboptimal for a population in which 20–30% have CNS involvement at baseline [41,42]. Crizotinib has now been functionally displaced by selective type Ib agents in jurisdictions where they are accessible (Table 1).

Type II MET inhibitors (cabozantinib, merestinib, glesatinib) bind the kinase in its inactive (DFG-out) conformation and have not been formally approved for METex14 NSCLC. Their relevance is principally in the resistance setting: D1228 and Y1230 mutations that confer resistance to type Ib drugs typically retain sensitivity to type II compounds [43,44,45]. This complementarity is the conceptual basis for sequential or combinatorial type I → type II strategies (Section 4.1.3).

#### 3.1.2. Type Ib—Selective MET TKIs (Current Standard of Care)

Type Ib inhibitors bind the active (DFG-in) conformation with high MET selectivity, yielding markedly improved therapeutic indices over multi-kinase agents. Two are FDA-approved for METex14-altered NSCLC: capmatinib (accelerated approval May 2020; converted to regular approval August 2022) [46] and tepotinib (accelerated approval February 2021; converted to regular approval February 2024) [47].

In the GEOMETRY mono-1 phase II trial, capmatinib produced an ORR of 68% (95% CI, 48–84%) with median DOR of 12.6 months in treatment-naïve patients with METex14-altered NSCLC, and ORR of 41% with DOR of 9.7 months in previously treated patients [16,48]. Intracranial activity was robust, with iORR exceeding 50% in evaluable patients with brain metastases [49]. Peripheral edema, nausea, and creatinine elevation are the dominant toxicities; the creatinine rise reflects inhibition of renal tubular transporters rather than true nephrotoxicity and stabilizes early without dose adjustment in most patients.

Tepotinib was approved on the basis of the VISION trial, which prospectively validated plasma ctDNA-based METex14 detection alongside tissue NGS [17]. Overall ORR was approximately 46% across cohorts, with concordance between tissue and plasma selection of about 70%—a paradigm-relevant finding because it permits treatment initiation when tissue is exhausted or non-diagnostic. Long-term VISION follow-up demonstrates a 2-year landmark PFS of approximately 25% [50]. Intracranial ORR of around 55% has been reported in patients with brain metastases [51]. Peripheral edema is the dominant adverse event, occurring in approximately 70% of patients and typically managed with diuretics, dose reduction, and close attention to fluid balance.

Savolitinib, approved in China for METex14-altered NSCLC (NMPA conditional approval 2021, converted to full approval in January 2025) on the basis of a single-arm phase II trial showing ORR of 49.2% [36], has been most influential globally as the MET partner in osimertinib combinations for EGFR-mutated, MET-amplified post-osimertinib disease (Section 5) [52,53].

#### 3.1.3. Next-Generation and Emerging MET TKIs

Next-generation MET TKIs are being developed with two principal goals: improved CNS penetration and activity against on-target D1228 and Y1230 resistance mutations [45,54,55]. Elzovantinib (TPX-0022), a macrocyclic MET/SRC/CSF1R inhibitor, demonstrated preliminary activity in MET TKI-pretreated patients in the SHIELD-1 phase 1/2 study, although clinical development has subsequently been deprioritized [54]. Glumetinib (SCC244), reported in the GLORY phase II Chinese study, produced ORRs in the range of 60–66% in treatment-naïve METex14 NSCLC with manageable toxicity [55]. Several other agents are in earlier-phase development, generally focused on biophysical properties (lipophilicity, lower molecular weight) that favor blood–brain barrier penetration and on alternative kinase-binding modes that may circumvent recurrent resistance mutations.

### 3.2. MET Antibodies and Antibody-Drug Conjugates

#### 3.2.1. Amivantamab

Amivantamab is a fully human IgG1 bispecific antibody that simultaneously engages the extracellular domains of EGFR and MET, promotes receptor degradation, and elicits antibody-dependent cellular cytotoxicity and phagocytosis through Fc-mediated immune effector recruitment [56,57]. Approved in EGFR exon 20 insertion-mutated NSCLC (PAPILLON, CHRYSALIS) and as part of post-osimertinib regimens (MARIPOSA-2), it occupies an expanding role in MET-driven contexts because of its dual EGFR + MET action [57,58]. Its established clinical role is thus within EGFR-mutant NSCLC—exon 20 insertion disease and the post-osimertinib setting, where MET-mediated resistance is one of several mechanisms it may address—rather than in pure METex14-driven or primary MET-amplified NSCLC, where its activity is considerably less established and it is not a standard option. We discuss it here for its MET-directed mechanism, not as a treatment for METex14 disease. The principal logistical limitation is intravenous administration—now partially mitigated by subcutaneous formulations—and the toxicity profile of infusion reactions, paronychia, rash, and edema.

#### 3.2.2. Telisotuzumab Vedotin (Teliso-V)

Telisotuzumab vedotin (Emrelis) received FDA accelerated approval on 14 May 2025 for adults with locally advanced or metastatic, non-squamous EGFR-wildtype NSCLC harboring high c-Met protein overexpression (defined as IHC 3+ staining in ≥50% of tumor cells using the VENTANA MET [SP44] assay) who have received at least one prior systemic therapy [59,60]. The pivotal phase II LUMINOSITY trial demonstrated an ORR of 35% (95% CI, 24–46%) with median DOR of 7.2 months in the c-Met-high cohort [59]. The agent is composed of an anti-c-Met monoclonal antibody conjugated to monomethyl auristatin E (MMAE) via a cleavable linker, enabling targeted cytotoxic delivery and a bystander-killing effect that is particularly relevant in heterogeneous tumors [61,62]. Peripheral neuropathy (MMAE-related), fatigue, and edema are the dominant toxicities, with dose modifications required in approximately 40% of patients. Biomarker standardization is an unresolved issue: the SP44 assay is the companion diagnostic, but H-score versus percentage 3+ staining metrics differ across platforms and predict response with imperfect concordance.

#### 3.2.3. Future ADC Innovations

Several next-generation MET-directed ADCs are in early clinical development. Topoisomerase I-payload constructs (analogous to deruxtecan-based ADCs in HER2 and TROP2) may achieve activity in c-Met-low tumors by virtue of bystander killing of antigen-negative neighboring cells. MET × EGFR bispecific ADCs are in preclinical development. Site-specific conjugation chemistry is improving DAR (drug-to-antibody ratio) homogeneity, which may translate to more predictable pharmacokinetics and reduced off-target toxicity.

### 3.3. Combination Strategies

#### 3.3.1. MET Inhibitors and Immunotherapy

The combination of MET TKIs with PD-1/PD-L1 checkpoint inhibitors has, on the whole, been disappointing in METex14-altered NSCLC. The biology underlying this disappointment is paradoxical and instructive: although METex14-altered tumors frequently express high PD-L1 (≥50% in approximately 41% of cases in the Sabari series), they typically have low tumor mutational burden, an inflammation-poor microenvironment, and—in retrospective series—an ORR to single-agent immune checkpoint inhibitors of only 17% with median PFS of 1.9 months [63]. PD-L1 expression in this context appears to be driven by oncogenic MET signaling rather than by an underlying T-cell-inflamed phenotype, which explains why a high PD-L1 score does not translate into clinical benefit. Adding hepatotoxicity concerns from MET TKI + checkpoint inhibitor combinations, the net assessment is that immune checkpoint blockade should not be considered a default partner for MET-directed therapy outside well-designed clinical trials. Some isolated responses have been reported in tumors with concurrent high PD-L1 and TMB, suggesting biomarker-selected combinations may warrant further investigation in carefully chosen subpopulations [63]. That said, the picture is more nuanced than uniform resistance. Durable responses to immune checkpoint inhibitors have been documented in a minority of METex14 patients—for example, a series of six cases with progression-free survival exceeding 18 months [64]—and a large real-world analysis found that benefit varied with smoking history, tumor mutational burden, co-mutation profile (notably TP53, which was associated with better immunotherapy outcomes), and treatment line, with chemoimmunotherapy and even checkpoint-inhibitor monotherapy yielding meaningful responses in selected (often older, comorbid ex-smoker) patients, even though PD-L1 expression did not reliably predict benefit [65]. Selected METex14 subsets may therefore still derive benefit from immune checkpoint inhibition, and this remains an area where the population is underrepresented in prospective biomarker studies.

#### 3.3.2. MET Inhibitors Plus EGFR Inhibitors in EGFR-Driven Resistance

MET amplification is among the most common acquired resistance mechanisms to osimertinib in EGFR-mutated NSCLC. Reported frequencies vary considerably (7–25% in published series, up to ~50% in heavily-selected cohorts) and depend strongly on the assay (FISH versus NGS), the threshold applied, and the line in which osimertinib was used [12,29]. The therapeutic logic of dual EGFR + MET blockade is straightforward: EGFR remains an active driver, and MET amplification engages PI3K and RAS signaling through GRB2/GAB1 scaffolding independent of EGFR phosphorylation [12].

This rationale has now been validated across three trials. The phase II SAVANNAH study demonstrated that savolitinib 300 mg twice daily plus osimertinib produced an ORR of 56% (95% CI, 45–67%) and median PFS of 7.4 months in the high-MET cohort defined by IHC 3+ in ≥90% of tumor cells and/or FISH GCN ≥ 10 [31]. Critically, lower MET cutoffs identified populations with markedly inferior outcomes (ORR 9%, mPFS 2.8 months in the IHC3+/≥50% but not ≥90% subgroup), illustrating that MET-amplification thresholds matter clinically and not just statistically. The phase III SACHI study, presented at ASCO 2025, confirmed superiority of savolitinib + osimertinib over platinum-based chemotherapy in MET-amplified post-EGFR TKI disease, with ORR 58% versus 34%, mPFS 8.2 versus 4.5 months, and significant CNS protection [66]. On the basis of SACHI, savolitinib plus osimertinib received full NMPA approval in China in June 2025; the combination is not approved in the United States or Europe, where confirmation from the global phase III SAFFRON trial is awaited. The phase II INSIGHT 2 trial similarly demonstrated activity of tepotinib + osimertinib in MET-amplified post-osimertinib NSCLC [32]. MARIPOSA-2 established amivantamab + chemotherapy (with or without lazertinib) as an unselected post-osimertinib option, with mPFS of 6.3 months versus 4.2 months for chemotherapy alone [58].

#### 3.3.3. MET Inhibitors and KRAS G12C Inhibitors

MET pathway activation has been identified as an emerging mechanism of acquired resistance to KRAS G12C inhibitors (sotorasib, adagrasib), occurring in approximately 5–10% of progression cases [67,68,69]. Preclinical work supports dual MET + KRAS G12C blockade in restoring sensitivity, and clinical combinations (e.g., adagrasib + capmatinib) are entering early-phase evaluation [70]. RAS pathway reactivation is, however, typically polyclonal—involving secondary KRAS mutations, NRAS/HRAS upregulation, MET, EGFR, and FGFR amplification, and PI3K activation—which means MET inhibition addresses only a subset of resistant clones [67]. Realistic expectations are warranted.

#### 3.3.4. DNA Damage Repair Inhibitors and MET Blockers

Preclinical data suggest that MET pathway activation is associated with upregulation of DNA repair pathways and that MET-driven epithelial-to-mesenchymal transition correlates with reduced homologous recombination efficiency [71]. Co-occurring STK11 and KEAP1 alterations, frequent in METex14 tumors, may further modulate susceptibility to PARP and ATR inhibitors [71]. This is a hypothesis-generating area; rigorous patient-selection biomarkers are not yet established.

## 4. Molecular Mechanisms of Acquired Resistance

**Figure 2:**
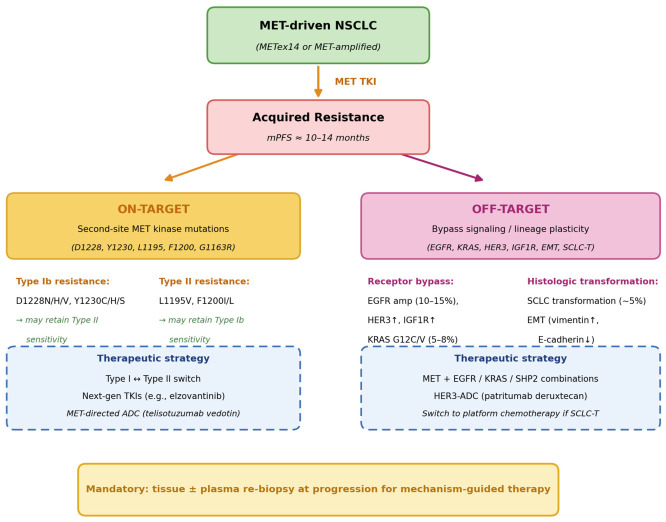
Mechanisms of acquired resistance to MET tyrosine kinase inhibitors and rational therapeutic strategies. On-target resistance involves second-site kinase domain mutations (D1228, Y1230 for type Ib drugs; L1195, F1200 for type II drugs), generally addressable by switching inhibitor class. Off-target resistance involves bypass receptor tyrosine kinase activation (EGFR amplification, HER3 upregulation, KRAS mutations), histologic transformation (SCLC transformation, EMT), or downstream pathway rewiring, requiring matched targeted combinations or class-switched chemotherapy. This schematic applies predominantly to METex14-driven and high-level MET-dependent tumors treated with selective MET TKIs; the on-target mutations shown (D1228, Y1230) are characteristic of that setting. Acquired resistance patterns—including the prevalence and biological relevance of these mutations—may differ in MET-amplified NSCLC, particularly where low-level or polysomic amplification does not represent true MET dependency. EMT, epithelial-to-mesenchymal transition; SCLC-T, small-cell lung cancer transformation.

Despite high initial response rates to selective MET TKIs, acquired resistance is essentially universal, with median PFS of 10–14 months across the major trials. This 10–14-month range applies chiefly to METex14 populations treated with selective MET TKIs; outcomes in MET-amplified NSCLC are considerably more heterogeneous and depend strongly on the amplification level, focality, assay methodology, and clinical context. Resistance mechanisms can be partitioned into on-target (kinase domain) mutations, off-target bypass signaling, and lineage plasticity, and the specific mechanism dictates rational subsequent therapy (Figure 2).

### 4.1. On-Target Resistance

#### 4.1.1. Second-Site Kinase Domain Mutations

Second-site MET kinase domain mutations represent the most well-characterized mechanism of acquired resistance to type Ib MET inhibitors. The two recurrent loci are D1228 (variants N, H, V, A) in the activation loop, and Y1230 (variants C, H, S) adjacent to the DFG motif [43,44,72]. D1228 mutations are identified in approximately 20–30% of post-capmatinib or post-tepotinib biopsies, while Y1230 mutations occur in 10–15% [43]. Less common solvent-front and gatekeeper-adjacent variants include G1163R (typically post-crizotinib), L1195V, and F1200I/L (Table 2).

#### 4.1.2. Structural Basis for Resistance

Crystallographic and computational modeling work has established the structural mechanisms underlying these mutations. D1228 normally forms a salt bridge with K1110 that helps stabilize the active kinase conformation favored by type Ib inhibitors; substitution to asparagine, histidine, or valine disrupts this network and reduces drug binding affinity [72]. The METD1228V mutation, originally identified in a patient progressing on savolitinib + osimertinib, was demonstrated through structural simulation to abolish the π–π interaction that anchors type Ib drugs while preserving the binding pocket geometry recognized by the type II inhibitor cabozantinib [72]. Y1230 substitutions disrupt aromatic stacking with type Ib drug aryl groups, again with relative preservation of type II binding [44]. Importantly, these mutations do not abolish kinase activity—MET remains constitutively active despite drug resistance, maintaining oncogenic signaling and confirming that MET dependency persists at progression.

#### 4.1.3. Type I Versus Type II Cross-Sensitivity

The complementary cross-sensitivity profile of type I and type II MET inhibitors is one of the more strategically useful findings in the field. Recondo and colleagues demonstrated in patient-derived data that two of three patients with on-target resistance mutations achieved partial responses upon switching between type I and type II inhibitors [43]. In vitro, D1228 and Y1230 variants generally retain sensitivity to cabozantinib and merestinib, whereas type II–selective resistance mutations (L1195V, F1200I/L) retain sensitivity to type Ib drugs [44,72]. This bidirectional asymmetry forms the rational basis for sequential or combinatorial strategies, although prospective clinical validation in large cohorts is still pending.

### 4.2. Off-Target Resistance

#### 4.2.1. Bypass Signaling Pathways

Off-target resistance involves activation of alternative receptor tyrosine kinases or downstream nodes that bypass continued MET inhibition. EGFR amplification, identified in 10–15% of post-MET TKI biopsies, restores RAS–MAPK and PI3K–AKT signaling and provides the rationale for MET + EGFR combinations at progression [43]. Acquired KRAS mutations (G12C, G12V) bypass MET inhibition through constitutive RAS-MAPK activation in approximately 5–8% of cases [43,73,74]. HER3 upregulation, particularly in tumors with concurrent EGFR alterations, activates NRG1–HER3–PI3K signaling and may be addressable by HER3-directed agents such as patritumab deruxtecan. IGF1R upregulation is a less frequent but mechanistically important bypass node that maintains PI3K–AKT signaling independent of RAS.

#### 4.2.2. Lineage Plasticity and Histologic Transformation

Histologic transformation, while less frequent than in EGFR-mutated NSCLC, has been documented in METex14-altered tumors. Small-cell transformation occurs in approximately 5% of cases progressing on MET TKI therapy and is enriched in tumors with concurrent TP53 and RB1 alterations [75,76]. These tumors lose MET dependency and require platinum-etoposide chemotherapy directed at small-cell biology rather than further MET inhibition. Epithelial-to-mesenchymal transition (EMT) represents a more common phenotypic change, with vimentin upregulation, E-cadherin loss, and broad TKI resistance, often without loss of MET expression. EMT is associated with metastatic propensity and may identify patients in whom upfront combinatorial strategies are appropriate.

#### 4.2.3. RAS-MAPK Pathway Rewiring

Beyond discrete genomic events, downstream signaling architecture can be rewired without new mutations. Upregulation of GAB1 and SHP2 scaffold proteins, dysregulated negative-feedback loops, and amplification of RAS-MAPK output enable sustained ERK activation despite MET inhibition. SHP2 inhibitors (RMC-4630, TNO155) are being evaluated in combination with MET TKIs as a strategy to interrupt this adaptive rewiring, with preliminary evidence of feasibility in early-phase studies.

## 5. MET Amplification as Acquired Resistance to Other TKIs

MET amplification has emerged as one of the leading—though not solely dominant—mechanisms of acquired resistance to osimertinib in EGFR-mutated NSCLC. Reported frequency varies widely (7–25% in most prospective series, up to ~50% in selected cohorts) and is heavily influenced by assay platform (FISH, NGS-based GCN, or IHC), threshold definition, and timing of the post-progression biopsy [12,29]. This heterogeneity is itself a clinical problem: cross-trial comparisons, registry-based prevalence estimates, and clinical decisions about who should receive an MET-targeted partner all depend on assay standardization that does not yet exist. Direct cross-trial comparison of SAVANNAH, SACHI, and INSIGHT 2 is correspondingly hazardous: each applied a different MET-amplification definition and assay (SAVANNAH, IHC3+/≥90% or FISH GCN ≥10; INSIGHT 2, FISH GCN ≥5; SACHI, centrally confirmed MET amplification), different selection stringency, and different comparators, so apparent differences in response rate and PFS across these studies partly reflect biomarker definition rather than true differences in drug activity.

Mechanistically, MET amplification bypasses osimertinib-mediated EGFR inhibition by activating PI3K–AKT and RAS–MAPK through GRB2/GAB1 scaffolding, independent of EGFR phosphorylation [12]. The implication is that EGFR signaling remains active and continued EGFR inhibition is required—dual blockade, not sequential monotherapy.

The clinical evidence supporting MET-targeted strategies in this setting has matured rapidly. The phase II SAVANNAH study established savolitinib 300 mg b.i.d. + osimertinib as an effective oral combination in patients with high MET expression (IHC3+/≥90% and/or FISH GCN ≥ 10), producing ORR 56%, mDOR 9.9 months, and mPFS 7.4 months [31]. The phase III SACHI study, the first randomized comparison of an oral MET-TKI combination versus chemotherapy in this setting, confirmed superiority for ORR (58% vs. 34%), mPFS (8.2 vs. 4.5 months), and CNS outcomes, providing a chemo-free option for patients with MET-amplified post-EGFR TKI progression [66]. This combination received full NMPA approval in China in June 2025; it is not yet approved in the United States or Europe pending the confirmatory global phase III SAFFRON trial. The phase II INSIGHT 2 study demonstrated similar activity for tepotinib + osimertinib, supporting a class effect of type Ib MET inhibitors combined with osimertinib [32]. MARIPOSA-2 established amivantamab + chemotherapy ± lazertinib as an unselected post-osimertinib regimen with PFS benefit over chemotherapy [58]; benefit is observed across subgroups but not specifically restricted to MET-amplified tumors, and combination toxicity is non-trivial (Table 3).

MET amplification as resistance to non-EGFR drivers is less common but biologically important. In post-sotorasib and post-adagrasib KRAS G12C-mutated tumors, MET amplification is identified in approximately 5% [67,68]. Rare MET amplification has been described in alectinib- and brigatinib-resistant ALK-rearranged NSCLC, although ALK kinase mutations dominate the resistance landscape in that setting [77,78]. The clinical implication, in all cases, is that comprehensive genomic profiling at progression—including assays capable of detecting copy-number changes—is essential.

## 6. Clinical Management of MET-Altered NSCLC in 2025

### 6.1. Diagnostic Considerations

Optimal diagnosis of MET alterations in 2025 requires recognition that the three actionable lesions—METex14, MET amplification, and c-Met overexpression—require different assays and define different patient populations. METex14 is best detected through paired DNA + RNA NGS, with RNA capturing cryptic splice variants that DNA panels with limited intronic coverage may miss [6,23]. MET amplification requires FISH (with explicit reporting of MET/CEP7 ratio and absolute GCN) or quantitative NGS-based copy-number analysis; ambiguous or borderline results should be reflexed to the alternative method. c-Met overexpression for telisotuzumab vedotin eligibility requires IHC using the FDA-approved VENTANA MET (SP44) RxDx assay, scored as IHC 3+ in ≥50% of tumor cells [60].

Liquid biopsy is now established as a complementary—not substitute—diagnostic. The VISION study prospectively validated plasma ctDNA-based METex14 detection with approximately 70% concordance with tissue [17,33]. Plasma genotyping is particularly useful when tissue is exhausted, when rapid turnaround is needed, and at progression for resistance-mechanism profiling [34]. False negatives can occur with shedding-poor tumors and predominantly intracranial disease, and absence of plasma detection should not preclude tissue confirmation in suspected cases. Detection of MET amplification in plasma is a particular challenge, distinct from METex14 and resistance-mutation detection: low-level amplification and its distinction from chromosome 7 polysomy are difficult to resolve in ctDNA, and concordance between tissue FISH and plasma NGS for amplification is imperfect and strongly influenced by tumor fraction and shedding characteristics. A negative plasma result for MET amplification is therefore weak evidence and should prompt tissue-based confirmation before a MET-amplified resistance mechanism is excluded. Recommended reporting standards should specify the assay used, the threshold applied (especially for amplification), the proportion of cells scored, and any limitations of the specimen.

### 6.2. Treatment Algorithms

**Figure 3:**
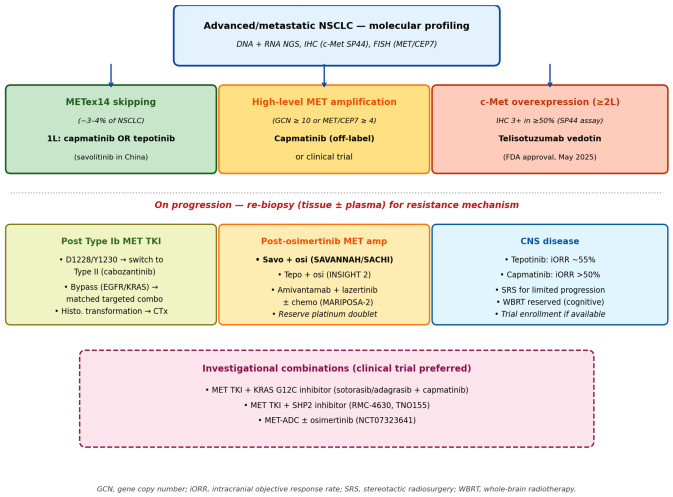
Proposed 2025 treatment algorithm for MET-altered non-small cell lung cancer. Three principal entry points define initial therapy: METex14 skipping mutations (selective type Ib TKI), high-level focal MET amplification (off-label MET TKI or trial enrollment), and c-Met protein overexpression in pretreated EGFR-wildtype non-squamous NSCLC (telisotuzumab vedotin). Re-biopsy at progression directs subsequent therapy according to the dominant resistance mechanism. This algorithm represents the authors’ evidence-informed proposal—an expert synthesis of the cited trial and biomarker data—rather than a formal practice guideline or consensus statement. Each entry point is supported by the regulatory approvals and trials referenced in Section 3 and Section 5, but availability is jurisdiction-dependent (for example, the savolitinib + osimertinib combination is approved only in China as of 2025, and frontline MET TKI use in primary high-level amplification remains off-label or investigational); treatment decisions should follow local approvals and multidisciplinary assessment. CTx, chemotherapy; GCN, gene copy number; iORR, intracranial objective response rate; SRS, stereotactic radiosurgery; WBRT, whole-brain radiotherapy.

First-line therapy for METex14-altered advanced NSCLC is a selective type Ib MET TKI—capmatinib or tepotinib (savolitinib in jurisdictions where approved) (Figure 3). Choice between agents is based on toxicity profile, dosing convenience, prescriber familiarity, and CNS disease, where both agents have demonstrated meaningful intracranial activity. Treatment should continue until progression or intolerable toxicity.

On progression, molecular re-biopsy is mandatory. Plasma ctDNA is the appropriate first step, with tissue biopsy reserved for confirmatory testing and for cases where plasma is non-diagnostic. Detection of D1228 or Y1230 mutations argues for trial enrollment with a next-generation MET TKI or—where feasible—a switch to a type II inhibitor. Bypass alterations (EGFR amplification, KRAS mutations, HER3 upregulation) point toward matched combination strategies. Histologic transformation—SCLC or EMT—argues for platform chemotherapy directed at the new histology.

Telisotuzumab vedotin is now an option in the second-line and later setting for c-Met-overexpressing, EGFR-wildtype, non-squamous NSCLC, regardless of METex14 status [59,60]. The agent’s mechanism is independent of kinase inhibition, providing a rational option in patients whose tumors retain MET protein expression after kinase inhibitor failure.

In MET-amplified post-osimertinib disease, the choice among savolitinib + osimertinib, tepotinib + osimertinib, and amivantamab-based regimens depends on the strength of MET amplification (high-cutoff biomarker selection favors oral combinations), CNS involvement, prior therapy, performance status, and route preference [31,32,58,66]. Platinum-doublet chemotherapy is reasonable but should not be the default given the activity of biomarker-matched options.

These algorithms are necessarily idealized relative to real-world practice, and several implementation barriers deserve explicit acknowledgment. Access to RNA-based NGS—needed for reliable METex14 detection—is uneven outside specialized centers; reimbursement for comprehensive genomic profiling and for MET-directed agents varies by payer and region [79]; and drug approvals differ markedly across jurisdictions (for example, the savolitinib + osimertinib combination is approved only in China), so a biomarker-matched option identified at testing may be locally unavailable. Repeat tissue biopsy at progression is frequently constrained by lesion accessibility, patient fitness, and procedural risk. In community and non-academic settings these constraints are amplified, and pragmatic compromises—single-platform testing, plasma-only genotyping, or empirical chemotherapy when matched agents are inaccessible—are common. The algorithm should therefore be read as an evidence-informed ideal to be adapted to local testing capacity, drug availability, and reimbursement.

### 6.3. CNS Disease Management

CNS disease is common at diagnosis and during treatment in MET-altered NSCLC. The CNS activity of capmatinib (iORR > 50%) and tepotinib (iORR ~55%) is well-documented and supports continued systemic therapy in patients with asymptomatic, non-progressive brain metastases [49,51]. Stereotactic radiosurgery (SRS) is preferred for limited symptomatic or progressing brain disease; whole-brain radiotherapy (WBRT) is reserved for diffuse leptomeningeal involvement or extensive brain disease in patients with limited systemic options, given the cognitive sequelae. Trial enrollment should be considered for CNS-dominant progression, as several next-generation MET TKIs prioritize improved blood–brain barrier penetration.

## 7. Discussion

The MET field in 2025 illustrates both the maturation and the remaining tensions of precision oncology in NSCLC. Maturation is evident in the regulatory landscape—two type Ib TKIs approved for METex14, a c-Met-directed ADC newly approved for c-Met-overexpressing disease, and a phase III randomized trial (SACHI) supporting biomarker-driven combination therapy in post-osimertinib MET amplification. Maturation is also evident in mechanistic understanding: the structural basis of D1228 and Y1230 resistance, the cross-sensitivity of type I and type II inhibitors, and the polyclonal nature of bypass resistance are now sufficiently well-characterized to permit rational sequencing rather than empirical drug-switching.

Tensions, however, persist. The most consequential is diagnostic. MET amplification thresholds vary across trials, FISH and NGS copy-number measurements are not interchangeable, and the SAVANNAH experience demonstrates that lower cutoffs identify populations with markedly diluted benefit. c-Met IHC scoring for ADC eligibility is platform-dependent, and the H-score versus percentage 3+ debate is unresolved. Until assay standardization is achieved, real-world treatment decisions will involve substantial uncertainty that the clinical trial literature does not fully reflect.

A second tension is the gap between strong evidence in MET-amplified post-osimertinib disease and the absence of approved combination regimens in this setting in most jurisdictions outside China. SACHI provides level-1 randomized evidence; SAFFRON, the confirmatory phase III, is in progress. On the strength of SACHI, savolitinib plus osimertinib became the first MET-directed combination approved in this setting (China NMPA, June 2025); approval elsewhere is not yet in place. Until regulatory approval extends to other jurisdictions, off-label use, expanded access, and trial enrollment will remain the principal pathways for biomarker-matched therapy.

A third tension concerns immunotherapy. The poor performance of MET TKI + checkpoint inhibitor combinations in METex14 NSCLC—despite frequent high PD-L1 expression—underscores that single biomarkers can mislead. PD-L1 driven by oncogenic signaling does not predict the same response as PD-L1 driven by an inflamed microenvironment, and treating these as interchangeable is a translational error that the field has now sufficiently demonstrated.

## 8. Future Directions

Several priorities define the path forward. First, next-generation MET TKIs designed to retain activity against D1228 and Y1230 mutations—and ideally with improved CNS penetration—remain a critical unmet need; existing type II inhibitors are imperfect substitutes given multikinase off-target toxicity. Second, MET-directed ADCs with alternative payloads (topoisomerase I inhibitors, bystander-active linkers) and bispecific configurations may extend benefit to c-Met-low and heterogeneous tumors that fail telisotuzumab vedotin. Third, rational triplet combinations—EGFR + MET + KRAS pathway modulators, or MET + SHP2—warrant systematic evaluation, with the caveat that toxicity will be the binding constraint. Fourth, ctDNA-based serial monitoring offers a path to detect resistance earlier than radiographic progression, potentially enabling pre-emptive treatment intensification or switching, although prospective evidence that pre-emptive intervention improves outcomes remains limited. Fifth, spatial heterogeneity of resistance—different mechanisms in different metastatic sites—argues for adaptive therapy strategies that may require multiple concurrent biopsies; the operational complexity of such approaches in routine practice is non-trivial. Sixth, MET-altered NSCLC remains underrepresented in immunotherapy biomarker studies; the assumption that high PD-L1 predicts response is now known to be false in this population, and rigorous evaluation of alternative correlates (TMB, gene expression signatures, T-cell infiltration) is needed. Seventh, dedicated CNS-focused trials—rather than retrospective intracranial subgroup analyses—are needed for an alteration with such high CNS tropism.

## 9. Conclusions

MET-targeted therapy in NSCLC has matured from an aspirational concept to a clinically operational framework defined by mutation class, drug class, and resistance mechanism. METex14 skipping is treated upfront with a selective type Ib TKI; high-level MET amplification is treated similarly when primary or with EGFR + MET combination therapy when acquired post-osimertinib; c-Met overexpression in pretreated EGFR-wildtype disease is now addressable with telisotuzumab vedotin. Across all three entry points, resistance is the unifying problem and re-biopsy at progression is the unifying solution. The 2025 evidence base supports biomarker-guided sequencing, but the field remains constrained by inconsistent diagnostic thresholds, incomplete CNS data, and limited approved options in the MET-amplified post-osimertinib space. Continued biomarker-driven trial design, particularly with attention to the high-cutoff MET amplification population that derives the largest benefit, will determine whether MET-targeted therapy continues to mature into a precise, sequenceable, and durable strategy.

## Figures and Tables

**Table 1:** Comparative summary of MET-directed therapies approved or in advanced clinical development for NSCLC.

Agent	Class	Pivotal Trial/Setting	Efficacy (METex14)	CNS Activity	Key Toxicities/Notes
Crizotinib	Type Ia	PROFILE 1001 (METex14)	ORR ~32%; mPFS 7.3 mo	Poor	GI, edema, QTc; superseded by selective TKIs
Capmatinib	Type Ib	GEOMETRY mono-1 (FDA accel. 2020; regular approval 2022)	1L: ORR 68%, mDOR 12.6 mo; 2L+: ORR 41%	iORR > 50%	Peripheral edema, nausea, increase in creatinine
Tepotinib	Type Ib	VISION (FDA 2021; full approval 2024)	ORR ~46%; 2-yr PFS ~25%	iORR ~55%	Peripheral edema (≈70%); plasma-NGS validated
Savolitinib	Type Ib	Phase II (China NMPA; cond. 2021, full approval 2025); not FDA/EMA-approved	ORR 49% (sarcomatoid-enriched cohort)	Limited data	Edema, nausea; combo with osimertinib in EGFRm
Cabozantinib	Type II	Off-label/preclinical	Active in vitro vs. D1228/Y1230	Penetrant	HFSR, hypertension, GI; multikinase off-target
Elzovantinib (TPX-0022)	Type Ia (next-gen)	SHIELD-1 (Phase 1/2)	Early signal in TKI-pretreated NSCLC	Penetrant (preclinical)	Dizziness; MET/SRC/CSF1R
Glumetinib (SCC244)	Type Ib (next-gen)	GLORY (Phase II, China)	ORR ~66% in METex14	Preclinical penetrant	Edema, transaminitis
Amivantamab	EGFR-MET bispecific Ab	MARIPOSA/MARIPOSA-2/PAPILLON	Active post-osi MET amp setting	Modest	Infusion reactions, rash, paronychia, edema; IV
Telisotuzumab vedotin	c-Met ADC (MMAE)	LUMINOSITY (FDA accel. May 2025)	ORR 35% in c-Met-high (3+/≥50%)	Limited published	Peripheral neuropathy, fatigue, edema

ADC, antibody-drug conjugate; CNS, central nervous system; DOR, duration of response; iORR, intracranial objective response rate; ORR, objective response rate; PFS, progression-free survival; mDOR, median duration of response.

**Table 2:** Major on-target acquired resistance mutations to selective MET tyrosine kinase inhibitors with structural mechanism and predicted cross-sensitivity.

Mutation	Frequency Post-Type Ib	Structural Mechanism	Predicted Sensitivity	Clinical Implication
D1228 (N/H/V/A)	20–30%	Disrupts salt bridge with K1110; loss of Type Ib H-bond network	Resistant: capmatinib, tepotinib, savolitinib. Retained: cabozantinib, merestinib (Type II)	Switch to Type II inhibitor
Y1230 (C/H/S)	10–15%	Loss of π–π stacking with Type Ib aromatic ring	Resistant: Type Ib. Variable Type II response (Y1230S retains Type II sensitivity)	Type II switch; ADC consideration
L1195V	<5%	Solvent-front; clashes with Type II hinge binders	Sensitive: Type Ib. Resistant: Type II	Maintain Type Ib
F1200I/L	<5% (Type II setting)	Gatekeeper-adjacent; impedes DFG-out binders	Sensitive: Type Ib. Resistant: Type II	Switch to Type Ib
G1163R	Rare (post-crizotinib)	Solvent-front substitution	Resistant: crizotinib. Retained: capmatinib, tepotinib, savolitinib	Switch from Type Ia to Type Ib

amp, amplification; H-bond, hydrogen bond; Type Ia: crizotinib; Type Ib: capmatinib, tepotinib, savolitinib; Type II: cabozantinib, merestinib, glesatinib.

**Table 3:** Pivotal trials of MET-directed strategies in EGFR-mutated NSCLC with MET-amplified acquired resistance to osimertinib.

Trial/Regimen	Phase/Setting	Population (Selection)	Key Outcome	Comment
**TATTON (savo + osi)**	Phase Ib	EGFRm post-EGFR-TKI; MET-amp/IHC+	ORR 33% post-3rd-gen TKI; 62–67% TKI-naïve	Established proof of principle
**SAVANNAH (savo + osi)**	Phase II	Post-1L osi; MET IHC3+/≥90% or FISH GCN ≥ 10	ORR 56% (BICR 55%); mPFS 7.4 mo; mDOR 9.9 mo	Defines threshold for benefit; high-cutoff selection critical
**SACHI (savo + osi)**	Phase III	Post-EGFR-TKI; MET-amplified	ORR 58% vs. 34% (chemo); mPFS 8.2 vs. 4.5 mo	First randomized chemo-free option in this setting
**INSIGHT 2 (tepo + osi)**	Phase II	Post-1L osi; MET-amplified (FISH GCN ≥ 5)	ORR 50% (TBx-selected); mPFS 5.6 mo	Confirms class effect of Type Ib + osi
**MARIPOSA-2 (ami + chemo ± laz)**	Phase III	Post-osi (unselected for MET)	mPFS 6.3 (ami + chemo) vs. 4.2 mo (chemo); HR 0.48	Approved option; benefit not MET-restricted
**CHRYSALIS-2 (ami + laz)**	Phase Ib	Post-osi, post-platinum	ORR 36% in chemo-experienced cohort	Oral lazertinib + IV amivantamab

ami, amivantamab; chemo, chemotherapy; FISH, fluorescence in situ hybridization; GCN, gene copy number; IHC, immunohistochemistry; laz, lazertinib; mDOR, median duration of response; mPFS, median progression-free survival; ORR, objective response rate; osi, osimertinib; savo, savolitinib; tepo, tepotinib.

## Data Availability

No new data were created or analyzed in this study. Data sharing is not applicable to this article.

## Abbreviations

ADC, antibody-drug conjugate; AGA, actionable genomic alteration; ALK, anaplastic lymphoma kinase; BICR, blinded independent central review; CBL, Casitas B-lineage lymphoma; CDKN2A/B, cyclin-dependent kinase inhibitor 2A/B; CNS, central nervous system; ctDNA, circulating tumor DNA; DAR, drug-to-antibody ratio; DDR, DNA damage response; DOR, duration of response; EGFR, epidermal growth factor receptor; EMT, epithelial-to-mesenchymal transition; FDA, U.S. Food and Drug Administration; FISH, fluorescence in situ hybridization; GCN, gene copy number; HGF, hepatocyte growth factor; IHC, immunohistochemistry; iORR, intracranial objective response rate; JM/JXD, juxtamembrane domain; METex14, MET exon 14 skipping; MMAE, monomethyl auristatin E; mPFS, median progression-free survival; NGS, next-generation sequencing; NSCLC, non-small cell lung cancer; NTRK, neurotrophic receptor tyrosine kinase; ORR, objective response rate; PD-L1, programmed death-ligand 1; PSC, pulmonary sarcomatoid carcinoma; RET, rearranged during transfection; ROS1, c-ros oncogene 1; SCLC, small-cell lung cancer; SH2, SRC homology 2; SRS, stereotactic radiosurgery; STAT, signal transducer and activator of transcription; TK, tyrosine kinase; TKI, tyrosine kinase inhibitor; TMB, tumor mutational burden; WBRT, whole-brain radiotherapy.
